# Structure-Based Engineering of a PTPsigma Ectodomain for Enhanced Solubility and Productivity

**DOI:** 10.3390/ijms26178345

**Published:** 2025-08-28

**Authors:** Sung Ho Park, Woochan Lim, Jian Kang, Sumin Jo, Hye Hyeon Jang, Heejin Yang, Suk Hyun Yoo, Myeongbin Kim, Seong Eon Ryu

**Affiliations:** Department of Bioengineering, College of Engineering, Hanyang University, Seoul 04673, Republic of Korea

**Keywords:** protein tyrosine phosphatase receptor sigma, structure-based engineering, solubility, production yield, therapeutic applications

## Abstract

Protein tyrosine phosphatase receptor sigma (PTPRS) regulates cellular signals involved in hematopoietic stem cell development, synaptic plasticity, and synovium differentiation. The soluble extracellular Ig-like domains of PTPRS have therapeutic potential by binding to a ligand, inhibiting the ligand-binding of endogenous PTPRS. However, the wild-type Ig-like domains have poor solubility, which limits their therapeutic use. In this study, we identified solvent-exposed hydrophobic residues on the surface of PTPRS and mutated the residues to hydrophilic residues for solubility-enhancing engineering. The mutagenesis screening increased its solubility up to five-fold. In addition, the expression yields were also increased by up to 14-fold. The biochemical and functional analysis of the engineered PTPRS showed that the mutant protein had comparable properties to the wild type. Thus, the engineered PTPRS has potential for therapeutic applications where modulation of PTPRS is critical.

## 1. Introduction

Protein tyrosine phosphatases (PTPs) dephosphorylate the tyrosine or serine/threonine residues of cellular regulatory proteins [1,2]. Protein tyrosine phosphatase receptor type S (PTPRS) regulates the regeneration of neural cells and hematopoietic stem cells, and synaptic plasticity [3,4,5]. PTPRS also promotes the invasiveness of synoviocytes [6]. PTPRS activity has been implicated in various diseases, including traumatic brain injuries, Alzheimer’s disease, stroke, multiple sclerosis, bone marrow damage, and rheumatoid arthritis [2,6,7,8,9,10,11,12]. The extracellular part (ectodomain) of PTPRS comprises three immunoglobulin (Ig)-like domains followed by nine fibronectin (FN-3) domains [13], where the N-terminal two Ig domains (PTPRSIg12s) bind to proteoglycans for PTPRS signaling [10,14,15]. The cytoplasmic part contains tandem PTP catalytic domains, with only the first exhibiting catalytic activity. The intracellular PTP catalytic activity is inhibited when two PTPRS molecules make dimers [16]. The binding of different proteoglycans to the extracellular Ig domains switches the dimerization status of the intracellular PTP catalytic domains, resulting in activity regulation [16,17]. Overexpression of chondroitin sulfate proteoglycans in diseased states in neural, hematopoietic, and synoviocytes promotes the monomeric state of PTPRS, resulting in the activation of the PTPRS catalytic activity.

The inhibition of PTPRS catalytic activity has the potential for therapeutic applications, and efforts to develop therapeutics targeting PTPRS have been reported [6,18,19]. The peptide resembling the wedge region of PTPRS, contributing to the dimer-induced activity inhibition, has therapeutic effects for stroke, multiple sclerosis, and spinal cord injury [7,10]. However, the inhibitory peptide must penetrate the membrane to reach the PTP catalytic domain in the cytosolic space. One of the allosteric inhibitors demonstrated promising results in promoting the recovery of bone marrow stem cells after radiation or chemotherapy-induced bone marrow damage in cancer patients [19]. However, the selectivity and potency of allosteric inhibitors need to be verified. The use of a soluble ectodomain of PTPRS, which inhibits PTPRS signaling in the extracellular space, is a way to circumvent difficulties caused by the peptide and small-molecule inhibitors of the catalytic domain. The soluble proteoglycan-binding ectodomain of PTPRS functions as a decoy for proteoglycan switch for the inhibition of invasiveness of synoviocytes in rheumatoid arthritis [6,20].

Here, we report the structure-based engineering of the PTPRS ectodomain-Fc fusion protein for enhanced solubility and production yield. For the therapeutic applications of proteins, poor solubility can cause difficulty in achieving therapeutic doses [21]. High protein concentrations are often necessary to obtain therapeutic effects by protein or antibody therapeutics [22]. The PTPRSIg12 protein has hydrophobic surface patches [13]. Thus, we designed and screened a series of mutations of the PTPRSIg12 protein that would not affect the proteoglycan binding. The screening resulted in mutant PTPRSIg12s with significant enhancement in solubility and production yield. The PTPRSIg12-Fc fusion protein with a solubility-enhanced PTPRSIg12 exhibited neural cell regeneration activity with high efficiency. The engineered protein has the potential for therapeutic applications in diseases related to the PTPRS signal pathway.

## 2. Results and Discussion

### 2.1. Structure-Based Residue Selection for Enhanced Solubility

**Figure 1:**
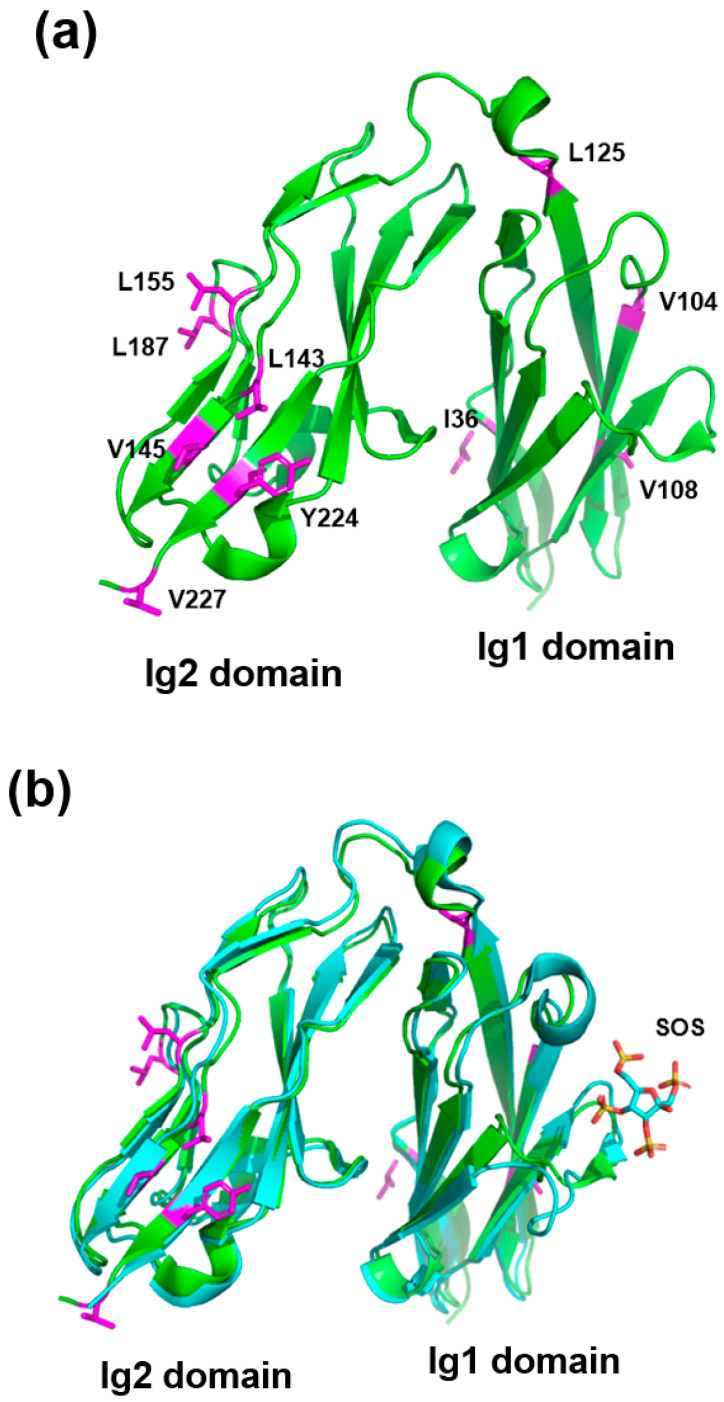
Structure-based mutant design. (**a**) Side chains of the structure-based mutation sites are represented on the structure of PTPRSIg12 (PDB code: 2YD2). (**b**) The complex structure (PDB code: 2YD8) between the PTPRS homolog PTPLAR and the chondroitin sulfate analog SOS (sucrose octasulfate) is superimposed on the structure of PTPRS (green). In (**a**,**b**), Ig1 and Ig2 domains are labeled.

The structure of PTPRSIg12 (PDB code: 2YD2) exhibits surface-exposed hydrophobic residues (Figure 1a). The surface hydrophobic residues can induce self-aggregation of PTPRSIg12 monomers, leading to the formation of large aggregates and precipitation in therapeutic concentrations. The role of the hydrophobic patches in PTPRSIg12 is not clear. The Fc fusion form of PTPRSIg12 (PTPRSIg12-Fc) was used in mouse model studies as a PTPRS decoy [6]. However, the same fusion protein exhibited a limited solubility and required detergents for high concentrations in our hands (unpublished results). We hypothesized that changes in the solvent-exposed hydrophobic side chain would enhance the solubility of the protein. Ten solvent-exposed hydrophobic residues were selected for the mutation candidates (Figure 1a). We chose solvent-exposed residues that do not overlap with the proteoglycan interaction sites (Figure 1b). Four residues (Ile 36, Val 104, Val 108, and Leu125) were in Ig1, and the other six residues (Leu 143, Val 145, Leu 155, Leu 187, Val 227, and Tyr 224) were in Ig2. Each residue was mutated to alanine or asparagine, and the solubility and production yield of the expressed proteins were measured for single or combined mutations (see below).

### 2.2. Solubility and Production Yield of Mutant Sets

**Figure 2:**
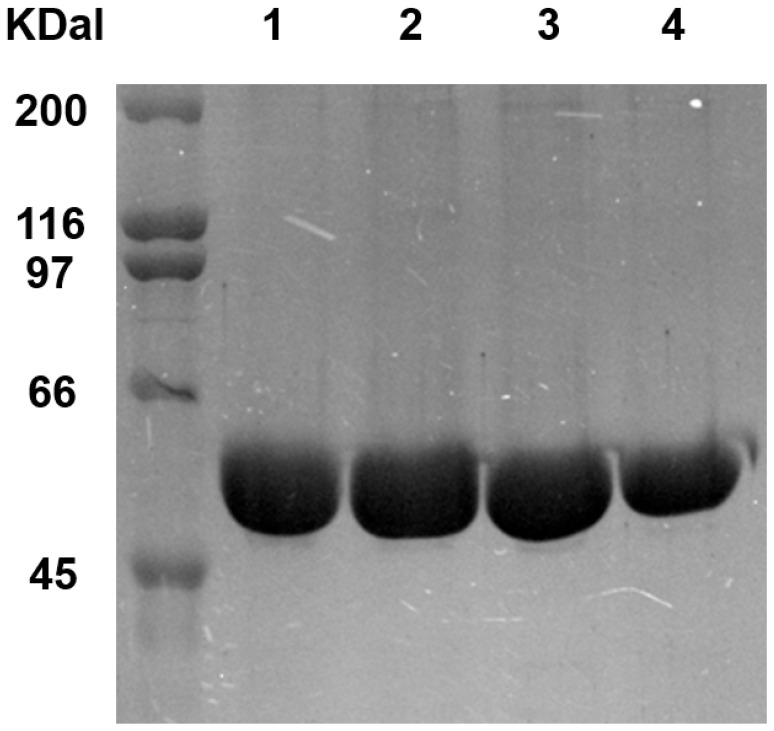
Purification of PTPRSIg12-Fc Coomassie-blue-stained SDS-PAGE gel of the wild-type and L155N/L187N mutant PTPRSIg12-Fc: lanes 1 and 2, the mutant (reduced); lanes 3 and 4, the wild type (reduced).

The wild-type and mutant PTPRSIg12 domains were fused with the immunoglobulin Fc for expression efficiency and therapeutic advantages [23]. The PTPRSIg12-Fc was expressed in CHO cells as a secreted protein. The secreted proteins were purified by Ni-NTA affinity column by using the C-terminal His-tag (see Section 4), and the purified protein was analyzed by SDS-PAGE (Figure 2). The expression yield was about 6 mg/L when the wild-type PTPRSIg12-Fc was cultured in a 25 mL volume. We assessed changes in the production yield and solubility of the structure-based mutations (see Section 4 and Table 1). In the table, the production yield of mutants was presented relative to that of the wild type. The relative solubility was presented as the ratio of proteins in the supernatants of the 400 mL sample to the 1.0 mL sample (see Section 4).

The L155N/L187A, L155N/L187N, and L143A/L145A/L155N/L187N mutants exhibited a relative solubility of 100%, indicating there was no precipitation loss in the concentration from the 1.0 mL to 400 mL volume (see Methods). Among these mutants, the production yields of the L155N/L187N and L143A/L145A/L155N/L187N mutants were significantly higher than that of the wild type (8.8- and 14.7-fold, respectively). We chose the L155N/L187N mutant for biochemical and functional characterization because it had less probability of structural perturbation due to mutations in only two sites. In addition, fewer mutation sites would be advantageous in minimizing unwanted immune responses in the human body in therapeutic uses of the variants. Most mutants with good solubility and expression yield consisted of mutations in Ig2. Only one mutant (I36A) in Ig1 exhibited a slight increase in expression yield. Interestingly, changes of hydrophobic residues to asparagine appear to be more effective than those to alanine (Table 1). The long hydrophilic side chain of asparagine may have contributed better than the simple truncation of hydrophobic side chains to a methyl group in alanine.

### 2.3. Ligand-Binding Property

**Figure 3:**
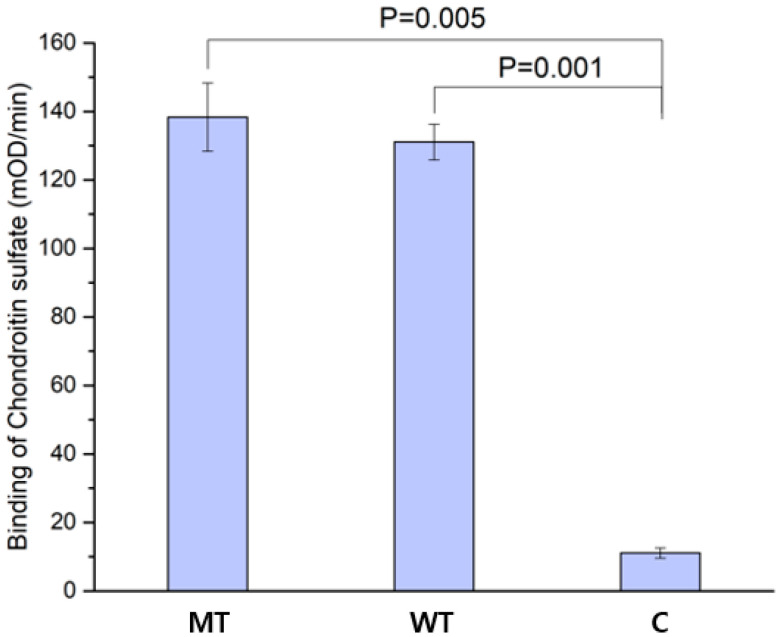
Chondroitin sulfate affinity. The chondroitin sulfate (CS)-binding affinity of the L155N/L187N mutant PTPRSIg12-Fc (MT) is compared with those of the wild-type PTPRSIg12-Fc (WT) and the control Fc (C). The error bars are calculated from the triplicate measurements. The experiment was performed with 4.0 μg/mL PTPRSIg12-Fc and 200 μg/mL CS (see Methods).

The ability of the L155N/L187N mutant to bind chondroitin sulfate (CS) was compared to that of the wild type by ELISA (Figure 3). Because CS would not bind to a 96-well plate easily, we chose to attach PTPRSIg12-Fc to the 96-well plate. The CS binding activity of the mutant was comparable to that of the wild type. In Figure 3, the affinity for the mutant was approximately 139, which was slightly higher than that of the wild type (approximately 135). The difference lies within errors that may have arisen from slight differences in measurement conditions. The comparable CS-binding activity of the mutant and wild type indicates that the mutant can be used as a decoy for the modulation of PTPRS signaling.

### 2.4. Neurite Outgrowth Activity

**Figure 4:**
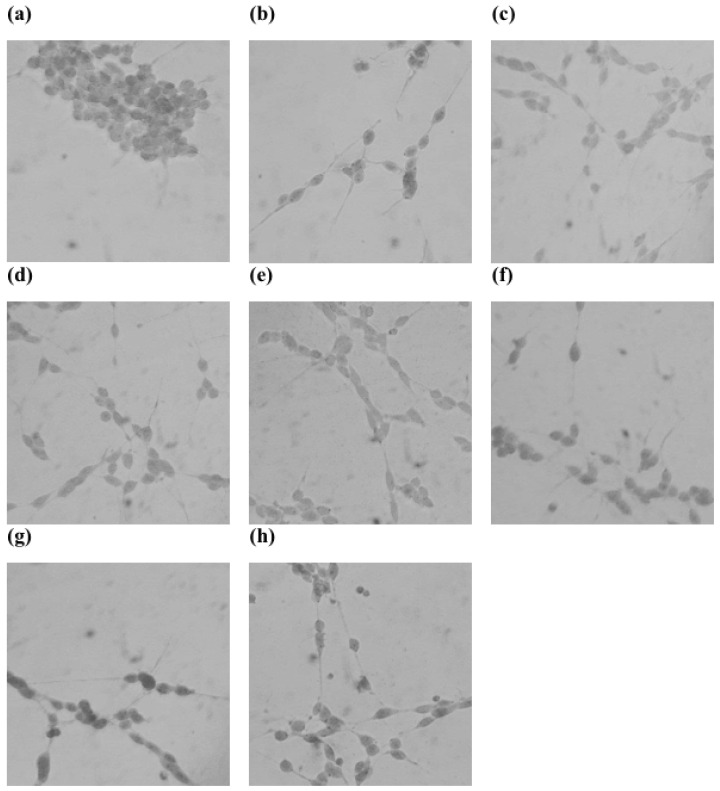
Representative images of neurite outgrowth of SH-SY5Y cells. (**a**) Non-treated SH-SY5Y cells. (**b**) RA^+^ SH-SY5Y cells. (**c**) RA^+^ CS^+^ SH-SY5Y cells. (**d**) RA^+^ CS^+^ and 50 nM wild-type-PTPRSIg12-Fc-treated SH-SY5Y cells. (**e**) RA^+^ CS^+^ 100 nM wild-type-PTPRSIg12-Fc-treated SH-SY5Y cells. (**f**) RA^+^ CS^+^ 50 nM L155N/L187N mutant-PTPRSIg12-Fc-treated SH-SY5Y cells. (**g**) RA^+^ CS^+^ 100 nM L155N/L187N mutant-PTPRSIg12-Fc treated SH-SY5Y cells. (**h**) RA^+^ CS^+^ 200 nM L155N/L187N mutant-PTPRSIg12-Fc-treated SH-SY5Y cells.

**Figure 5:**
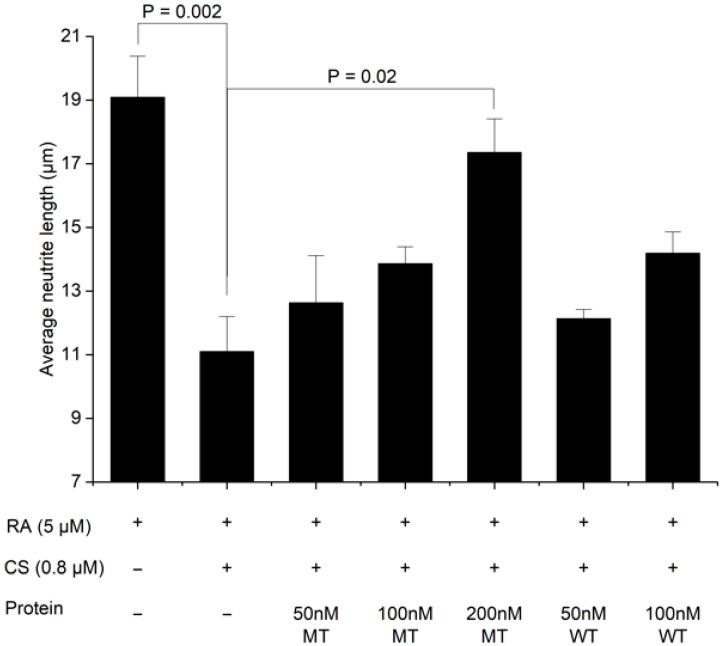
Average neurite length. Neurite outgrowth assay results are displayed for different reagent treatment conditions. Average neurite length for different RA, CS, and PTPRSIg12-Fc conditions is presented. The L155N/L187N mutant and wild-type PTPRSIg12-Fcs are indicated as MT and WT, respectively.

A cell assay was performed using the L155N/L187N mutant. Neuronal SH-SY5Y cells, which have PTPRS in their cell membrane to regulate neuronal differentiation [24], were used to evaluate the efficacy of the wild-type and mutant PTPRSIg12-Fc proteins. PTPRS signaling occurs by the interaction of the Ig12 domain of PTPRS with chondroitin sulfate (CS). The CS-mediated PTPRS signaling downregulates the differentiation of neuronal cells in traumatic neuronal injury and stroke [7,10]. Thus, we sought to evaluate the wild-type and mutant PTPRSIg12-Fc proteins as a therapeutic by inhibiting the PTPRS:CS interaction that occurs in the extracellular space. To analyze the neurite outgrowth promotion activity of PTPRSIg12-Fc, we measured the mean length of neurites of SH-SY5Y cells (Figure 4 and Figure 5). The mutant PTPRSIg12-Fc revealed superior activity in comparison to the wild type at a high dose, where the wild-type protein is not soluble. In the activity assay, the treatment of CS to SH-SY5Y cells inhibited RA-induced neurite formation. In the addition of the L155N/L187N mutant PTPRSIg12-Fc protein to the CS-inhibited cells, the inhibition was reversed by about 80% ((17.5 − 11)/(19 − 11) = 81) of the original neurite formation (Figure 5). However, the wild type was able to reverse the activity only by about 60% ((19 − 14)/(19 − 11) = 62) due to limited solubility. The wild-type protein precipitated at concentrations above 100 nM. In comparison, the L155N/L187N mutant was completely soluble at 200 nM and above. In addition, the higher production yield of the mutant protein (Table 1) would provide another advantage for the protein’s therapeutic use.

## 3. Conclusions

By using a structure-based design approach, we produced solubility-enhanced variants of the PTPRS ectodomain. The soluble ectodomain has been implicated as a decoy for ligand–receptor interactions in PTPRS signaling, with potential therapeutic applications in diseases such as cancer, Alzheimer’s disease, stroke, traumatic brain injury, and rheumatoid arthritis. In therapeutic applications, the wild-type protein is not suitable due to its poor solubility, whereas solubility-enhanced variants can achieve the protein concentration required for therapeutic efficacy. We proved the cellular activity of a variant in a neuronal regeneration assay. With an appropriate combination of brain-delivery technology, including brain-shuttle antibody fusions [25], the solubility-enhanced variants can be used for neuronal regeneration in Alzheimer’s disease, stroke, and other brain diseases. For application in non-brain diseases, the Fc-fused form can be used. Thus, the solubility-enhanced PTPRS variants have potential for development as therapeutic agents. The solubility-enhanced variants may induce an unwanted immune response in the human body, which must be closely monitored in the early stages of clinical study.

## 4. Methods

### 4.1. Protein Expression and Purification

PTPRSIg12, comprising residues 30-231 of PTPRS, was inserted into a lab-made human Fc-containing vector based on pcDNA3.1/MycHis-A (Invitrogen, Waltham, MA, USA). The Fc-containing vector had been created by inserting the human IgG1 heavy chain signal sequence and residues 221–447 of the Fc region into the pcDNA vector. PTPRSIg12 was inserted between the signal sequence and the Fc region to secrete the PTPRS12-Fc protein into the culture medium. The mutant PTPRSIg12-Fc proteins were generated using a Quickchange site-directed mutagenesis kit (Stratagene, La Jolla, CA, USA) The wild-type and mutant PTPRSIg12-Fc proteins were expressed in expiCHO-S (Thermo Fisher Scientific, Waltham, MA, USA). ExpiCHO cells were maintained in ExpiCHO Expression Medium (Gibco, Waltham, MA, USA) in a humidified 8% CO_2_ incubator at 37 °C. Media were supplemented with antibiotic–antimycotic (100×) (Sigma, Burlington, MA, USA). ExpiCHO cells were thawed or subcultured at a density of 0.15 × 10^6^ cells/mL–0.3 × 10^6^ cells/mL and were grown at a density of 4 × 10^6^ cells/mL–6 × 10^6^ cells/mL and diluted to 3 × 10^6^ cells/mL–4 × 10^6^ cells/mL. After cells were cultured to a concentration of 7 × 10^6^–10 × 10^6^ cells/mL, they were diluted to 6 × 10^6^ cells/mL for transfection.

ExpiCHO transfections were conducted in 125 mL Erlenmeyer cell culture flasks (Corning) using an ExpiCHO Expression System Kit (Thermo Fisher Scientific, Waltham, MA, USA) according to the ExpiCHO expression system user guide. Briefly, 100 µL of plasmid DNA (0.2 mg/mL) and 80 µL of ExpiFectamine CHO Reagent (Thermo Fisher Scientific, Waltham, MA, USA) were diluted in OptiPRO SFM medium (Thermo Fisher Scientific, Waltham, MA, USA)up to 1.0 mL. Diluted ExpiFectamine CHO reagent and plasmid DNA were mixed and incubated for 1–5 min. The ExpiFectamine CHO/plasmid DNA mixtures were then added to the 25 mL of cultured cells. For ExpiCHO Standard Protocol transfections, 150 µL of ExpiCHO enhancer and 6.0 mL of ExpiCHO Feed were added 18~22 h post-transfection. After 8–10 days of transfection, the transfection supernatants were harvested by centrifuging at 4000× *g* for 10 min at 4 °C and then filtered through a 0.45 µm cellulose ester membrane filter (Advantech, Cincinnati, OH, USA). The fusion protein was purified by nickel-affinity chromatography using NI-NTA resin (Qiagen, Venlo, The Netherlands) using the C-terminal His-tag.

### 4.2. Structure-Based Design

The structures were displayed using the program Pymol (Schrodinger) and manually inspected to estimate the degrees of solvent exposure of side chains. Among the solvent-exposed hydrophobic residues, those with more than 70% solvent exposure were selected as potential mutation sites. Among the solvent-exposed hydrophobic residues, we further selected residues whose mutations would not introduce structural perturbation on the chondroitin-sulfate binding site. For this, we avoided residues whose main chain and side chain atoms are within 5 Å distance from the atoms of the chondroitin–sulfate interaction residues.

### 4.3. Solubility Measurement of Mutants

The solubility of each mutant was estimated by measuring the amount of protein that was retained in solution during a step-wise concentration. The concentration of purified protein was determined by a DC protein assay kit (Bio-Rad, Hercules, CA, USA). The protein solution was centrifuged at 14,000 rpm for 4 min at 4 °C to separate the precipitated fraction from the soluble part. To quantify the production yield, the protein elution fractions (8.0 mL) of the purification column were concentrated to 1.0 mL. The precipitate during the concentration was removed by centrifugation. The concentration of the supernatant was measured. For the quantification of solubility, the above 1.0 mL concentrated solution was further concentrated to 400 mL. The protein concentration of the supernatants in the 1.0 mL and 400 mL samples was measured. The amount of protein in the solution was calculated by multiplying the concentration by the volume of the solution. The relative solubility was estimated by the ratio of the amount of protein in the 400 mL and 1.0 mL samples. The experiments were triplicated, and the average values are presented in Table 1.

### 4.4. Ligand-Binding Assay

The chondroitin sulfate (CS) binding was estimated in a protein-A-coated 96-well plate (Thermo Fisher Scientific, Waltham, MA, USA). The protein-A-coated 96-well plate was washed three times with PBS (0.137M NaCl, 0.0027M KCl, 0.01M Na2HPO4, and 0.0018M KH2PO4) before the wild-type or mutant PTPRSIg12-Fc proteins or the Fc control in 0.05% PBST (PBS with 0.05% Tween)+ 5% skim milk was added to the wells and incubated for 1 h. CS (Sigma, Burlington, MA, USA) was biotinylated by incubating 560 μL of 2.0 mg/mL CS and 140 μL of 10 mM Ezlink-sulfo-NHS-LC-LC-biotin (Thermo Fisher Scientific) at 37 °C for 3 h. Free biotin in the reaction mixture was removed by a Zeba desalting spin column in PBS. The biotinylated CS solution was added to the PTPRSIg12-Fc-protein-A well and incubated at room temperature for 1 h. The wells were washed 10 times with PBS. Streptavidin-HRP in 0.05% PBST was added and incubated for 2 h. After seven washes, TMB was added, and absorbance was measured at 650 nm every 2 min.

### 4.5. Neurite Outgrowth Assay

The neuronal SH-SY5Y cells were obtained from the Korea Cell Line Bank. The cells were maintained in Dulbecco’s modified Eagle’s medium (DMEM) supplemented with 10% (*v*/*v*) FBS (Gibco, Waltham, MA, USA) and antibiotic–antimycotic (GenDEPOT, Baker, TX, USA) (100×) at 37 °C and 8% humidified conditions. Pre-warmed trypsin-EDTA was added to cells at a confluency of 70% to 80%, and the cells were incubated at 37 °C for 2 min. The isolated cell and medium were transferred to a 15 mL conical tube and centrifuged at 300× *g* for 5 min at room temperature. The medium supernatant was removed by pouring, and the cell was resuspended in pre-warmed (37 °C) wash medium (PBS). Cells were centrifuged at 300× *g* for 5 min at room temperature. The medium supernatant was carefully discarded by pouring, and the cells were resuspended with warm wash medium for the second wash. The cell suspension was centrifuged at 300× *g* for 5 min at room temperature. Following the second wash, the supernatant was removed, and the cells were resuspended in differentiation medium (RPMI 1640, 1% FBS, 1% antibiotic–antimycotic). Collagen-coated 96-well plates were used to differentiate SH-SY5Y cells. Cells were seeded at 5–10 × 10^3^ cells/well in plates and incubated at 37 °C, in an 8% CO_2_, humidified condition. An amount of 5 µM of retinoic acid (RA), 1 µM chondroitin sulfate (Sigma, Burlington, MA, USA), and PTPRSIg12-Fc were added to the medium in order to validate the differentiation. Two days after differentiation, the medium was eliminated by pipetting, and 20 µL of Neurite Stain Solution (Millipore, Burlington, MA, USA) was added for cell staining. After 4 min, the photo was taken, and the mean neurite extension length was measured from the photo by using the ImageJ (version 1.53m) program [26].

## Figures and Tables

**Table 1:** Production yield and relative solubility.

Mutant	Production Yield	Relative Solubility (%)
Wild type	1.0	21
L125A	0.7	30
I36A	1.5	21
L155A	1.9	44
Y224A	1.3	34
Y224A/V227A	1.0	43
L155A/L187A	1.1	34
L155A/L187A/Y224A	3.2	66
L155A/L187A/Y224A/V227A	0.6	93
L155N/L187A	1.3	100 (111) *
L155N/L187N/Y224A	11.3	79
L143A/L155A/L187A/Y224A/V227A	7.9	34
L155N/L187A/Y224A	6.2	73
L155N/L187A/Y224A/V227A	5.9	67
L143A/L155A/L187A	8.1	89
L155N/L187N	8.8	100 (115)
L143A/L145A/L155N/L187N	14.7	100 (101)

* Values in parentheses were the original values, which were rounded to 100. The values above 100 may be due to sample handling and measurement errors.

## Data Availability

Data will be available on request.

## References

[1] Ohtake Y., Saito A., Li S. (2018). Diverse functions of protein tyrosine phosphatase sigma in the nervous and immune systems. Exp. Neurol..

[2] Stanford S.M., Bottini N. (2023). Targeting protein phosphatases in cancer immunotherapy and autoimmune disorders. Nat. Rev. Drug Discov..

[3] Wang Z.C., Gao Q., Shi J.Y., Guo W.J., Yang L.X., Liu X.Y., Liu L.Z., Ma L.J., Duan M., Zhao Y.J. (2015). Protein tyrosine phosphatase receptor S acts as a metastatic suppressor in hepatocellular carcinoma by control of epithermal growth factor receptor-induced epithelial-mesenchymal transition. Hepatology.

[4] Li H., Wong C., Li W., Ruven C., He L., Wu X., Lang B.T., Silver J., Wu W. (2015). Enhanced regeneration and functional recovery after spinal root avulsion by manipulation of the proteoglycan receptor PTPsigma. Sci. Rep..

[5] Ko J.S., Pramanik G., Um J.W., Shim J.S., Lee D., Kim K.H., Chung G.Y., Condomitti G., Kim H.M., Kim H. (2015). PTPsigma functions as a presynaptic receptor for the glypican-4/LRRTM4 complex and is essential for excitatory synaptic transmission. Proc. Natl. Acad. Sci. USA.

[6] Svensson M.N.D., Zoccheddu M., Yang S., Nygaard G., Secchi C., Doody K.M., Slowikowski K., Mizoguchi F., Humby F., Hands R. (2020). Synoviocyte-targeted therapy synergizes with TNF inhibition in arthritis reversal. Sci. Adv..

[7] Lang B.T., Cregg J.M., DePaul M.A., Tran A.P., Xu K., Dyck S.M., Madalena K.M., Brown B.P., Weng Y.L., Li S. (2015). Modulation of the proteoglycan receptor PTPsigma promotes recovery after spinal cord injury. Nature.

[8] Poirier A., Picard C., Labonte A., Aubry I., Auld D., Zetterberg H., Blennow K., Tremblay M.L., Poirier J., the PREVENT-AD research group (2024). PTPRS is a novel marker for early Tau pathology and synaptic integrity in Alzheimer’s disease. Sci. Rep..

[9] Li Y., Liu J., Huang J., Wei C., Ge L., Chung M., Zhu B., Guo Z., Zheng T., Li H. (2024). Reduced PTPRS expression promotes epithelial-mesenchymal transition of Schwann cells in NF1-related plexiform neurofibromas. Cancer Lett..

[10] Luo F., Wang J., Zhang Z., You Z., Bedolla A., Okwubido-Williams F., Huang L.F., Silver J., Luo Y. (2022). Inhibition of CSPG receptor PTPsigma promotes migration of newly born neuroblasts, axonal sprouting, and recovery from stroke. Cell Rep..

[11] Davis T.B., Yang M., Schell M.J., Wang H., Ma L., Pledger W.J., Yeatman T.J. (2018). PTPRS Regulates Colorectal Cancer RAS Pathway Activity by Inactivating Erk and Preventing Its Nuclear Translocation. Sci. Rep..

[12] Biersmith B.H., Hammel M., Geisbrecht E.R., Bouyain S. (2011). The immunoglobulin-like domains 1 and 2 of the protein tyrosine phosphatase LAR adopt an unusual horseshoe-like conformation. J. Mol. Biol..

[13] Coles C.H., Shen Y., Tenney A.P., Siebold C., Sutton G.C., Lu W., Gallagher J.T., Jones E.Y., Flanagan J.G., Aricescu A.R. (2011). Proteoglycan-specific molecular switch for RPTPsigma clustering and neuronal extension. Science.

[14] Shen Y., Tenney A.P., Busch S.A., Horn K.P., Cuascut F.X., Liu K., He Z., Silver J., Flanagan J.G. (2009). PTPsigma is a receptor for chondroitin sulfate proteoglycan, an inhibitor of neural regeneration. Science.

[15] Coles C.H., Mitakidis N., Zhang P., Elegheert J., Lu W., Stoker A.W., Nakagawa T., Craig A.M., Jones E.Y., Aricescu A.R. (2014). Structural basis for extracellular cis and trans RPTPsigma signal competition in synaptogenesis. Nat. Commun..

[16] Chien P.N., Ryu S.E. (2013). Protein tyrosine phosphatase sigma in proteoglycan-mediated neural regeneration regulation. Mol. Neurobiol..

[17] Lee S., Faux C., Nixon J., Alete D., Chilton J., Hawadle M., Stoker A.W. (2007). Dimerization of protein tyrosine phosphatase sigma governs both ligand binding and isoform specificity. Mol. Cell. Biol..

[18] Gardner R.T., Wang L., Lang B.T., Cregg J.M., Dunbar C.L., Woodward W.R., Silver J., Ripplinger C.M., Habecker B.A. (2015). Targeting protein tyrosine phosphatase sigma after myocardial infarction restores cardiac sympathetic innervation and prevents arrhythmias. Nat. Commun..

[19] Zhang Y., Roos M., Himburg H., Termini C.M., Quarmyne M., Li M., Zhao L., Kan J., Fang T., Yan X. (2019). PTPsigma inhibitors promote hematopoietic stem cell regeneration. Nat. Commun..

[20] Doody K.M., Stanford S.M., Sacchetti C., Svensson M.N., Coles C.H., Mitakidis N., Kiosses W.B., Bartok B., Fos C., Cory E. (2015). Targeting phosphatase-dependent proteoglycan switch for rheumatoid arthritis therapy. Sci. Transl. Med..

[21] Ebrahimi S.B., Samanta D. (2023). Engineering protein-based therapeutics through structural and chemical design. Nat. Commun..

[22] Garidel P., Kuhn A.B., Schafer L.V., Karow-Zwick A.R., Blech M. (2017). High-concentration protein formulations: How high is high?. Eur. J. Pharm. Biopharm..

[23] Duivelshof B.L., Murisier A., Camperi J., Fekete S., Beck A., Guillarme D., D’Atri V. (2021). Therapeutic Fc-fusion proteins: Current analytical strategies. J. Sep. Sci..

[24] Nunes-Xavier C.E., Zaldumbide L., Mosteiro L., Lopez-Almaraz R., Garcia de Andoin N., Aguirre P., Emaldi M., Torices L., Lopez J.I., Pulido R. (2021). Protein Tyrosine Phosphatases in Neuroblastoma: Emerging Roles as Biomarkers and Therapeutic Targets. Front. Cell Dev. Biol..

[25] Niewoehner J., Bohrmann B., Collin L., Urich E., Sade H., Maier P., Rueger P., Stracke J.O., Lau W., Tissot A.C. (2014). Increased brain penetration and potency of a therapeutic antibody using a monovalent molecular shuttle. Neuron.

[26] Schneider C.A., Rasband W.S., Eliceiri K.W. (2012). NIH Image to ImageJ: 25 years of image analysis. Nat. Methods.

